# Pancreaticoduodenectomy Is the Best Surgical Procedure for Zollinger–Ellison Syndrome Associated with Multiple Endocrine Neoplasia Type 1

**DOI:** 10.3390/cancers14081928

**Published:** 2022-04-11

**Authors:** Weihua Kong, Max Benjamin Albers, Jerena Manoharan, Joachim Nils Goebel, Peter Herbert Kann, Moritz Jesinghaus, Detlef Klaus Bartsch

**Affiliations:** 1 Department of Surgery, Philipps-University, 35041 Marburg, Germany; max_benjamin.albers@med.uni-marburg.de (M.B.A.); jerena.manoharan@uk-gm.de (J.M.); bartsch@med.uni-marburg.de (D.K.B.); 2 Department of Gastroenterology, Division of Endocrinology and Diabetology, Philipps-University, 35043 Marburg, Germany; joachimnils.goebel@uk-gm.de (J.N.G.); kannp@med.uni-marburg.de (P.H.K.); 3 Department of Pathology, Philipps-University, 35043 Marburg, Germany; moritz.jesinghaus@uni-marburg.de

**Keywords:** MEN1, ZES, gastrinoma, surgery, pancreaticoduodenectomy

## Abstract

**Simple Summary:**

Approximately 30% of patients with multiple endocrine neoplasia type 1 (MEN1) develop the Zollinger–Ellison syndrome (ZES), caused by solitary or multiple duodenal gastrinomas. Its management, especially regarding indication, timing, and type of surgery, is highly controversial. Therefore, the present study evaluated the long-term outcomes of pancreaticoduodenectomy (PD) versus non-PD resections in MEN1-ZES with regard to biochemical cure and quality of life. We found in a series of 35 patients that initial PD is the superior surgical procedure for MEN1-ZES, leading to long-term cure in about 80% of patients, fewer duodenopancreatic reoperations and an acceptable quality of life. Based on the results of this study, MEN1-ZES should be considered a surgically curable disease.

**Abstract:**

Aim: The aim of this research was to evaluate the long-term outcome of pancreaticoduodenectomy (PD) versus other duodenopancreatic resections (non-PD) for the surgical treatment of the Zollinger–Ellison syndrome (ZES) in patients with multiple endocrine neoplasia type 1 (MEN1). Methods: Prospectively recorded patients with biochemically confirmed MEN1-ZES who underwent duodenopancreatic surgery were retrospectively analyzed in terms of clinical characteristics, complications, cure rate, and long-term morbidity, including quality of life assessment (EORTC QLQ-C30). Results: 35 patients (16 female, 19 male) with MEN1-ZES due to duodenopancreatic gastrinomas with a median age of 42 (range 30–74) years were included. At the time of diagnosis, 28 (80%) gastrinomas were malignant, but distant metastases were only present in one (3%) patient. Eleven patients (31.4%) underwent pancreatoduodenectomy (PD) as the initial procedure, whereas 24 patients underwent non-PD resections involving duodenotomy with gastrinoma excision, enucleation of the pNEN from the head of the pancreas, and peripancreatic lymphadenectomy, either with or without distal pancreatectomy (i.e., either Thompson procedure, *n* = 12, or DUODX, *n* = 12). There was no significant difference in perioperative morbidity and mortality between the two groups (*p* ≥ 0.05). One (9%) patient of the PD group required reoperation for recurrent or metastatic ZES compared to eight (22.8%) patients of the non-PD resection groups. After a median follow-up time of 134 months (range 6–480) nine of 11 (82%) patients in the PD group, two of 12 (16%) patients in the Thompson procedure group, and three of 12 (25%) patients in the DUODX group had normal serum gastrin levels. In addition, the global health QoLScore was better in the PD group (76.9) compared to the Thompson procedure (57.4) and DUODX (59.5) groups. Conclusions: Initial PD seems to be the superior surgical procedure for MEN1-ZES, resulting in a long-term cure rate of about 80%, fewer duodenopancreatic reoperations, and an acceptable quality of life.

## 1. Introduction

Multiple endocrine neoplasia type 1 (MEN1) is a rare autosomal dominant hereditary tumor syndrome characterized by synchronous or metachronous occurrence of tumors, primarily of the parathyroid glands, the endocrine pancreas and duodenum, and the pituitary gland. Predisposition to MEN1 is determined by germline mutations in the *MEN1* gene, a tumor suppressor gene on the long arm of chromosome 11 (11q13) that codes for the protein menin [1,2]. Autopsy and clinical data revealed duodenopancreatic neuroendocrine neoplasms (dpNEN) in about 70–85% of MEN1-patients [3,4,5,6]. Approximately 30% of patients with dpNEN develop Zollinger–Ellison syndrome (ZES), which is almost exclusively caused by one or more duodenal gastrinomas [5,6,7]. In spite of a high rate of malignant spread to regional lymph nodes in 60–90% of patients, gastrinomas usually progress slowly [7,8,9,10]. However, about 20% of patients develop an aggressive growth with hepatic metastases [9,11]. Beside thymic carcinoid, gastrinomas and non-functioning dpNEN are the leading causes of disease-related death in MEN1 patients [3,12]. Currently, neither clinical nor molecular genetics can reliably distinguish aggressive and less aggressive phenotypes at the time of diagnosis [10,11,13].

The therapy for MEN1-ZES is still highly controversial regarding the indication and extent of surgery [14,15], since the prognosis is excellent in approximately 75% of MEN1 patients, indicating long-term survival, even without operation. Some experts do not consider MEN1-ZES a surgically curable disease and therefore advocate a non-surgical approach using proton pump inhibitors (PPIs) to control the symptoms of hypergastrinaemia [3,15,16]. It has been shown that 90% of patients with MEN1-ZES without pancreatic tumors on imaging are alive 15 years after the diagnosis, and even with diffuse distant metastases the 10-years survival rate is around 54% [17,18]. When taking into account, however, the young average age of diagnosis of MEN1-ZES (around 40 years), one might argue that an overall survival of 15–20 years, resulting in a significant number of patients dying before the age of 60, is not a reasonable goal in modern healthcare. Therefore, other groups indicate surgery whenever a pNEN reaches the size of 2 cm [17], which is a proven risk factor for the development of liver metastasis [19,20]. From today’s point of view, the majority of patients in the above studies most likely had duodenal gastrinomas and the pancreatic “gastrinomas” were nonfunctioning (NF) pNENs [18]. Nevertheless, an imageable pNEN of ≥1–2 cm in MEN1-ZES could be a useful surrogate parameter to indicate surgery, based on the good long-term survival of up to 90% at 10 years in this situation [17].

It is agreed that in order to cure hypergastrinemia and reduce the risk of distant metastasis, any surgery must include explorative duodenotomy or even resection of the entire duodenum in combination with radical periduodenopancreatic lymphadenectomy [14]. Performing a pancreaticoduodenectomy (PD) with a higher chance of cure is an option at the time of biochemical diagnosis of ZES, even in the absence of imageable tumors, and should be discussed with the patient [14,18,21,22]. There are several reasons for this strategy. First, PD allows for complete removal of the target organ due to hereditary predisposition, and long-term cure is then possible. This is the case in PD, since about 95% of gastrinomas in MEN1 arise from preceding proliferative gastrin cells in the duodenal mucosa in the duodenum [5,7,21]. Second, as part of the PD, the radical excision of the peripancreatic lymph nodes and pNEN in the head of the pancreas is performed, thus clearing the whole gastrinoma triangle. Although PD provides a cure rate of 60–100% of MEN1-ZES in small series [16,21,22,23,24,25,26,27], this procedure might have an increased perioperative mortality and morbidity and lead to reduced quality of life compared to non-PD resections in the long term. Therefore, the present study evaluated the long-term outcomes of PD versus non-PD resections in MEN1-ZES with regard to biochemical cure and quality of life.

## 2. Materials and Methods

Demographic and clinical data of patients with MEN1 have been collected in a prospective database at the Department of Surgery, Philipps-University Marburg since 1997. This database was approved by the local ethics committee. All patients provided written informed consent to register their data. Although the current research was part of routine clinical care, additional ethical approval was requested for the present study.

Patients with MEN1-ZES were retrieved from this database and their clinical data were retrospectively analyzed with special regard to demographics, clinical features, operative procedures, pathologic findings, and long-term follow-up, including quality of life. Some aspects of the clinico-pathological results of 15 patients were reported already in 2004 and 2013 [21,28].

The diagnosis of MEN1 was based on detailed personal and family history and/or the identification of a germline-mutation in the MEN1 gene. The mutation analysis of the MEN1 gene was performed using Taq cycle sequencing as described previously [29].

The diagnosis of gastrinoma was established by clinical symptoms, an elevated fasting serum gastrin level (≥125 pg/mL) together with low pH (<2) in the stomach, and a positive immunohistochemistry for gastrin in the tumor(s). A secretin stimulation test was performed until 2016 and was defined as positive if the serum gastrin concentration has increased to ≥200 pg/mL. Malignancy was defined by the strict criteria of infiltrating growth, lymph node, or distant metastases.

All MEN1 patients who met the criteria for gastrinoma underwent laparotomy after diffuse liver metastases were excluded by preoperative imaging. Preoperative imaging routinely comprised the following: thin-sectioned abdominal computed tomography (CT) and/or magnetic resonance imaging (MRI); somatostatin-receptor imaging with either scintigraphy (SRS) (prior to January 2014) or G68-Dotatoc-PET/CT (post-January 2014); or endoscopic ultrasonography (EUS). Selective arterial stimulation injection angiography (SASI) was performed from 1997 until 2010 in selected patients [30].

Surgery was indicated for all MEN1 patients with biochemically proven Zollinger–Ellison Syndrome who did not have severe comorbidities excluding duodenopancreatic surgery. Bidigital palpation of the pancreas and intraoperative ultrasonography was performed in all patients. Before 1997, the procedure of choice was duodenotomy with excision of duodenal gastrinomas with lymphadenectomy in the gastrinoma triangle and enucleation of pNENs out of the pancreatic head, either with (Thompson procedure [19]) or without distal pancreatectomy (DUODX). The Thompson procedure was performed when pNENs ≥ 1 cm were visualized on preoperative imaging in the pancreatic body or tail. Since 1997, we have recommended a PD procedure (resection of the pancreatic head and the duodenum and complete lymphadenectomy in the gastrinoma triangle including lymph node station 16) to patients without significant comorbidity. However, the advantages and disadvantages as well as the very low level of evidence were thoroughly discussed with each patient. Thus, the patients’ preference had a strong impact on the choice of surgical procedures in some cases. A total pancreatectomy was never indicated as the initial operation, since the authors consider this procedure an overtreatment. Moreover, none of the procedures were performed laparoscopically, since the authors do not consider the laparoscopic approach an adequate procedure for MEN1-ZES. The post-operative and long-term outcomes of MEN1 patients who underwent initial PD-resections (pancreaticoduodenectomy) were compared to those with non-PD resections, performed via either the Thompson or DUODX procedures.

Tumors were classified according to the current WHO classification at the time of diagnosis. [31]. Complications were scored according to the Clavien–Dindo classification [32] and pancreatic fistulas were graded according to the International Study Group of Pancreatic Fistula (ISGPF) [33].

Abdominal reoperations were performed in selected patients for recurrent ZES, newly developed functioning pNENs, or non-functioning pNENs ≥ 2 cm in the pancreatic remnant, or for metastases. A reoperation was only indicated in cases of a positive imaging result and a localized pattern of disease. Reoperative cases comprised enucleation of the tumor(s) in the pancreatic head or neck, distal pancreatic resection, completion pancreatectomy, duodenotomy with tumor excision, PD, or resection of metastases alone.

Cure of ZES was defined as a normal fasting gastrin concentration (<125 pg/L) at the time of hospital discharge after the initial operation and at the last follow-up investigations. The follow-up was based on the most recent in-hospital screening examination or obtained by the patient’s personal physician. The long-term results for insulin dependent diabetes mellitus were also recorded at the most recent follow-up.

Long-term quality of life (QoL) was assessed according to the European Organisation for Research and Treatment of Cancer Core Quality-of-Life questionnaire (EORTC QLQ-C30, version 3.0) [34]. All questionnaires were filled in by the patients between December 2020 and December 2021, either during a mailing survey or during the most recent screening visit. This self-administered instrument has been proven to be valid, discriminant, reliable, and internally consistent [35]. Reference QoL scores have been established from a random sample of the general population [36] as well as cancer patients [37]. Its 30 items incorporate a global health scale and QoL scale, 5 functional scales (physical, role, cognitive, emotional, social functioning) and 9 symptom scales (fatigue, pain, nausea, vomiting). All scores range between 0 and 100. Higher scores in the global scale and functional scales represent better QoL, whereas lower scores in the symptomatic scales are superior.

It has to be mentioned that the long-term assessment of insulin-dependent diabetes as well as the QoL score is based on an intention-to-treat analysis. This means that these results were influenced by the fact that some patients with an initial Thompson or DUODX procedure underwent subsequent duodenopancreatic reoperation(s) for recurrent ZES or metastases. However, four patients who ended up with total pancreatectomy after 1 to 4 reoperations were excluded from this analysis.

Descriptive and explorative statistics were performed using medians and ranges. Univariate analysis of clinical variables was tested by t test, Mann–Whitney U test, and analysis of variance (ANOVA), as appropriate. Because of the limited patient numbers, only the PD resection and non-PD resection groups were statistically compared (i.e., the Thompson procedure and DUODX procedures were combined for this analysis). The following patient characteristics were compared, using Fisher’s exact test for non-parametric data and the Mann–Whitney U test for parametric data: age at initial operation; number and type of tumors; rate of malignancy; the operative procedure performed; the cure rates for patients with duodenopancreatic gastrinomas who underwent either PD or non-PD resections; and the scores for quality of life. Kaplan–Meier survival curves with log rank analysis were calculated for overall survival and disease-free survival. All statistics were performed using the 28.0.1.0 SPSS and Stata 14.2 software. A value of *p* < 0.05 was considered to indicate statistical significance.

## 3. Results

Thirty-five patients (16 female, 19 male) with a median age of 42 (30–74) years were operated on for MEN1-ZES between April 1987 and May 2021. Of those, 11 (31.4%) patients initially had a PD, either pylorus-preserving (*n* = 6) or not (*n* = 5). Another 24 (68.6%) patients had non-PD resections involving a duodenotomy with excision of all tumors of the entire duodenum, a periduodenopancreatic lymphadenectomy, and enucleation of all pNENs in the head of the pancreas, either with (Thompson procedure, *n* = 12) or without distal pancreatectomy (*n* = 12, DUODX). In 32 (91.4%) patients, MEN1 was confirmed by identification of a MEN1-gene germline mutation. A total of 33 patients underwent surgery for primary hyperparathyroidism prior to ZES surgery; none of the patients had hypercalcemia at the time of ZES operation. The preoperative median serum gastrin level was 425 (range 160–44,095) pg/l with no significant differences between patients who underwent PD or non-PD resections (*p* = 0.974). A total of 26 (74%) patients had visualized dpNEN on imaging, three (8%) had suspected lymph node metastases, and one (3%) patient had liver metastases, with no significant differences observed between the PD and non-PD resection groups. The preoperative clinical data are summarized in Table 1.

A total of 12 patients (37%) experienced postoperative relevant complications (Clavien–Dindo ≥ 3), which required reoperation in eight (23%) patients. Major complications (*n* = 12) included postoperative pancreatic fistulas (POPF) type B (*n* = 1) and type C (*n* = 2), postoperative bleeding (*n* = 4), postoperative duodenal perforation (*n* = 1), postoperative abscesses (*n* = 2), and postoperative pancreatitis (*n* = 2). The complication rate was similar in PD, Thompson, and DUODX groups. The perioperative mortality rate was zero (Table 2). Overall, 30 of 35 patients had postoperatively normal fasting gastrin levels: 10 (91%) in the PD group, 10 (83%) in the Thompson, and 10 (83%) in the DUODX groups. A postoperative insulin-dependent diabetes occurred in none of the patients in any group in the first 30 postoperative days.

Histopathological examination of the specimens identified median three duodenal gastrinomas with no differences between the PD and non-PD groups. The number of resected pNENs was lower in the DUODX group (median 1) compared to the Thompson (median 3) and PD (median 4) groups. It was also of note that the number of resected lymph nodes was also lower in the DUODX group (median 9) compared to the Thompson (median 14) and PD (median 15) groups. Twenty-seven patients (77.2%) had malignant gastrinomas with the presence of lymph node (*n* = 26) and liver (*n* = 1) metastases at the time of surgery.

The long-term follow-up interval was comparable between patients with PD and non-PD resections (median 134 vs. 125 months), although it was significantly shorter in patients with DUODX (median 69 months, Table 3). At the most recent follow-up, 14 of 35 (40%) patients had normal serum gastrin levels without evidence of ZES. The long-term biochemical cure rate was significantly higher in the PD group (nine of 11, 82%) compared to the Thompson (two of 12, 16%) and the DUODX (three of 12, 25%) groups (*p* = 0.001). The number of patients who had reoperations for recurrent ZES was lower in the PD (one of 11, 9%) than in the non-PD groups (eight of 24, 66%). In the DUODX group, one patient required one reoperation (Thompson procedure), one patient required two reoperations (DUODX and completion pancreatectomy) and two patients required three reoperations that resulted in total pancreatectomy. In the Thompson group, two patients required one reoperation (DUODX), one patient required two reoperations (DUODX and residual pancreatectomy) and one patient underwent three reoperations (2x DUODX and 1x resection of the pancreas body with lymph node dissection).

**Figure 1:**
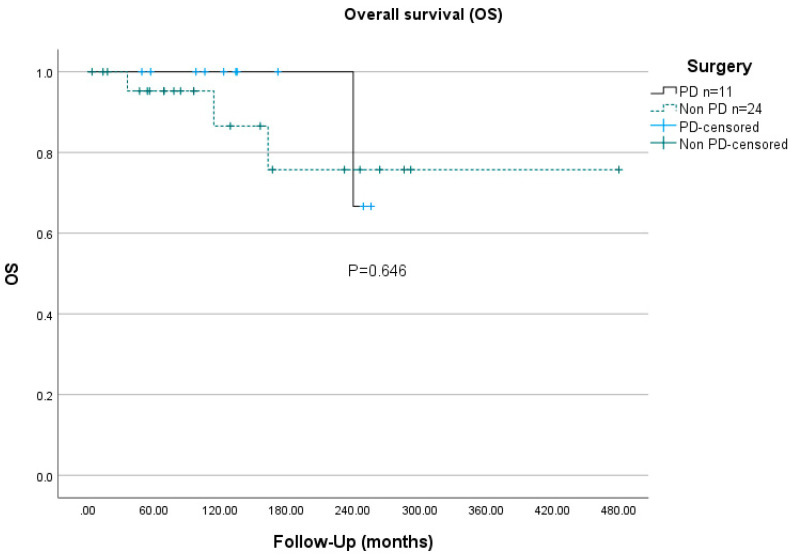
Overall disease survival (OS) of PD resections vs. non-PD resections for MEN1-ZES.

**Figure 2:**
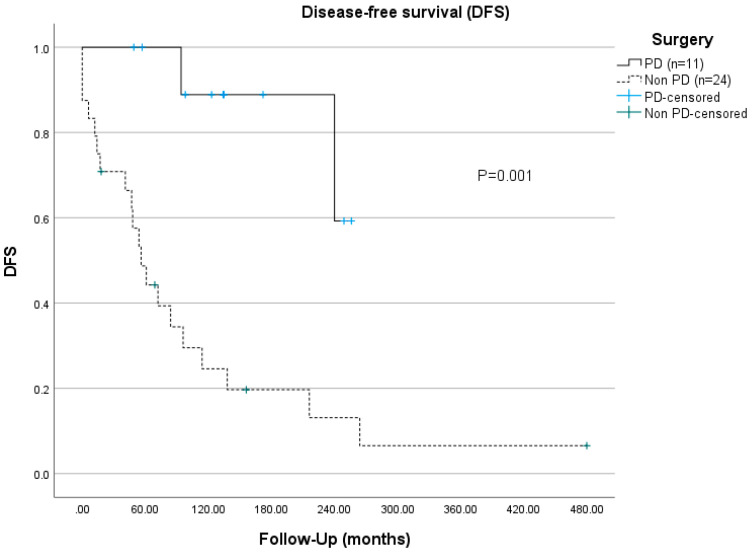
Disease-free survival (DFS) of PD resections (group 1) vs. non-PD resections (group 2/3).

At the most recent follow-up, none of the patients were deceased of ZES, but four (11%) patients had died of unrelated causes, including metastatic thymic carcinoid, metastatic gastric NEC, myocardial infarction, and liver metastasis of a colon carcinoma. None of the included MEN1 patients developed thymic NEN before the initial ZES operation. Three MEN1 patients suffered thymic NEN at ages 52, 53, and 54, a long time after the ZES diagnoses was made. The actuarial overall 10-years survival rates of MEN1-ZES were 90.9% for the PD group and 87.5% for the non-PD group (*p* = 0.646, Figure 1). The disease-specific 10-years survival rate was not statistically significantly different between the two groups (PD 100% vs. non-PD 92%). However, the disease-free survival rate was significantly longer in the PD group (134 vs. 55 months), as was the 5-years ZES-disease-free survival rate, with 100% compared to 55.5% in the non-PD group (*p* = 0.001, Figure 2).

Long-term follow-ups were conducted a median of 134 months following the initial operation and 96 months after the last ZES operation. At follow-up, 29 patients responded to the QoL questionnaire, four patients were deceased, and two patients (one PD, one with DUODX) declined to participate. Four patients were excluded from the analysis due to completion pancreatectomy (three patients with initial DUODX, one patient with initial Thompson procedure). The global health/QoL score was better in patients with initial PD resections (76.9) compared to those with an initial Thompson (57.4) or DUODX (59.5) procedure. The score for PD patients was also better in four functional scales (physical, role, cognitive, social functioning) as well as most of the symptom scales, including fatigue, pain, nausea and vomiting, appetite loss, financial difficulties, and insomnia (Table 4). The only symptom scale that was worse in patients with initial PD compared to initial DUODX was diarrhea (37.0 vs. 23.8). The global health/QoL scores and other scales in patients with initial PD did not differ from those of the general population (*p* = 0.853, global), except for the symptoms of diarrhoea (*p* = 0.019), and emotional functioning (*p* = 0.028). The analysis of patients who had only one duodenopancreatic resection for any MEN1-associated dpNEN also showed similar results between PD and non-PD resections (Table 4). None of the differences between PD and non-PD resections in either ZES or dp-NEN were statistically significant.

## 4. Discussion

This present study is one of the largest to compare short and long-term outcomes, including quality of life, between pancreaticoduodenectomy (PD) and different non-PD (Thompson and DUODX) resections in patients with MEN1-ZES. Although PD is potentially associated with a higher ZES cure rate, a major concern regarding PD as a primary surgical procedure is the potentially higher rate of complications as compared to non-PD resections. This fear is based on the fact that young MEN1 patients have a soft pancreatic tissue and often a small diameter (<3 mm) of the pancreatic and biliary ducts, which are proven risk factors for anastomotic complications [24,25,26,28,38]. In the presented series, the rate of patients with clinically relevant complications (Clavien–Dindo ≥ 3) was 36% (four of 11) after PD, which is in line with the reported 26% (eight of 31), 37% (6/16) and 64% (nine of 14) from PD series conducted in France, Italy, and the Netherlands [16,25,39]. The most frequent complication after PD in MEN1 is POPF type B and C occurring in 18% of the present series, and in 16.1% of the French [16], 27% of the Italian [25] and 50% of the Dutch series [39]. Postoperative hemorrhage was also a frequent complication in the French and Dutch PD series, occurring at 16% and 21%, respectively, but did not occur in the present cohort. It is of note, however, that the rate of clinically relevant complications in the present series did not differ between PD (four of 11, 36%) and non-PD resections (eight of 24, 33.3%). This complication rate for non-PD resections for MEN1-dpNEN is in the same range of 26% to 58% reported in previous studies [17,39,40,41]. The overall mortality of all procedures was zero in the present cohort, but perioperative mortality up to 4% has been reported in other series of PD resections for MEN1-dpNEN [16,39]. Regarding this, it must be emphasized that most MEN1-ZES patients undergoing PD are young (median 42 years in the present series), with an excellent physical condition to overcome even severe complications.

As reported earlier, MEN1-ZES is caused by one or multiple duodenal gastrinomas [7], which are malignant in a high proportion of 60–80% [8,9,10], and in 77.2% in our series. The rate of liver metastases at the time of diagnosis was slightly lower in our series (one of 35, 2.8%) compared to the proportions of 7.1% to 8% in other series [12,22]. This might be caused by the annual routine screening program and our relatively aggressive attitude to indicating surgery for MEN1-ZES [12,21,38].

The long-term biochemical cure rate of MEN1-ZES after median 147 months was significantly higher in the PD group (9/11, 82%) than in the non-PD group (5/24, 21%, *p* = 0.007). This high cure rate after PD supports other small previous series reporting cure rates of 61% to 100% after a median follow-up of up to 151 months (see Table 5). In contrast, non-PD resections such as the Thompson procedure or DUODX provide a maximum cure rate of 30%, and reached only 2% in the current series, when considering a normal fasting serum gastrin level after at least 5 years postoperatively [17,19,38]. The disease-free survival time was also significantly longer in the PD group than in the non-PD group (134 vs. 55 months, *p* = 0.001). This difference is not surprising, since only PD provides the complete removal of the ZES target organ duodenum [4,31,42,43] along with its proliferative gastrin cells that are a precursor in this genetically determined disease [8]. Furthermore, a PD procedure allows clearance of the whole gastrinoma triangle by a more radical dissection of periduodenopancreatic lymph nodes and possibly relevant NENs of the pancreatic head. In the present study, pathological examination revealed no significant difference in the number of duodenal gastrinomas and resected lymph nodes after PD than non-PD resections (see Table 2). Therefore, our data suggest that the more important factor for long-term cure might be the removal of the target organ duodenum, precluding local recurrence. Additionally, concomitant pNENs ≥ 2 cm in the remaining pancreas can be enucleated. Based on these cure rates, the statement of a former expert practice guideline [14] that ZES in MEN1 is a surgically incurable disease should be reconsidered.

The overall and the disease-specific 10-years survival of the PD-group and non-PD-group were 100% vs. 92% and 90.9% vs. 87.5%, respectively, and did not significantly differ (Figure 1). However, these 10-years overall survival rates are both certainly longer than in non-surgically managed patients with MEN1-ZES, ranging from 25% to 82% depending on the fasting gastrin level at the time of ZES diagnosis [21]. In the series of the French GTE group, the probability of death for MEN1-ZES was calculated to be 9.7% at 8 years after diagnosis [44]. One possible reason for better survival rates after surgery is that an early operation for MEN1-ZES, independent from the type of duodenopancreatic procedure, prevents the development of distant metastatic disease, which is the most life-threatening factor [45,46].

Another important point to consider is that patients with initial PD resection required less duodenopancreatic reoperations for recurrent ZES (9% vs. 33%) or other newly developed pNENs (9% vs. 12.5%) during long-term follow-up. A previous study reported that 16% (eight of 49) of patients required completion duodenopancreatectomy at median 8 years after an initial Thompson procedure for MEN1-ZES because of recurrent pNEN and hypergastrinemia [47]. An extended initial operation, either PD or distal pancreatectomy, was not a contraindication for reoperations. In our cohort, four patients had completion pancreatectomy. From a technical point of view, it is even easier to perform a left pancreatic resection after PD than a PD after a distal pancreatectomy. We have previously published on our experience with duodenopancreatic reoperations in MEN1 [48].

While PD might be the optimal procedure for patients with MEN1-ZES with regard to the high chance of cure, long-term quality of life and morbidity also have to be considered carefully. The rates of endocrine insufficiency with insulin-dependent diabetes mellitus as well as the rates of exocrine insufficiency were not different between PD (27% and 46%, respectively) and non-PD (36% and 42%, respectively) in the present study after a follow-up of more than 12 years (Table 3). The MEN1-PD studies from France and the Netherlands reported similar rates for long-term insulin-dependent diabetes mellitus (22% and 26%, respectively) and for exocrine pancreatic insufficiency (16% and 64%, respectively) [16,22].

Since patients with MEN1 are rarely cured from their pNENs disease in the long term as long as pancreatic tissue is in the body, the effect of duodenopancreatic operations on QoL and morbidity may be—other than survival—the most critical endpoints. Unfortunately, studies on the QOL of MEN1 patients in general or with regard to special topics are almost completely lacking. One recent cross-sectional study was performed using the national Dutch MEN1 cohort to evaluate Health Related Quality of Life based on the SF36 questionnaire in 227 genetically confirmed MEN1 patients [49]. The health-related QoL scores were significantly lower for most subscales in comparison with the general Dutch population. One other study focused on the QoL of MEN1 patients who underwent pancreaticoduodenal surgery for dpNEN [50]. The global QoL of 50 MEN1 patients evaluated with the EORTC-C30 questionnaire after duodenopancreatic surgery (70.3) did not significantly differ from the reference population (75.7) in most functional and symptomatic scales. This present study is the first to focus on the long-term QoL of MEN1 patients who underwent duodenopancreatic surgery for ZES. Overall, the global QoL score of MEN1-ZES patients was lower (64.6) than in a random sample of the general population (75.7) [36], but higher than in a random sample of cancer patients (61.3) [37]. It has to be emphasized, however, that the global QoL score was similar in the PD group (76.9) and the non-PD groups, either Thompson procedure (57.4) or DUODX (59.5), and even closer to that of the general population [36]. This also holds true if one compares MEN1 patients who had only one duodenopancreatic resection, either PD or non-PD, during their disease (Table 4). In addition, the scores of the PD group in the five functional scales (physical, role, emotional, cognitive, and social functioning) and the three symptom scales (fatigue, nausea and vomiting, and pain) as well as all other items did not differ from the non-PD groups (Table 4). Although duodenopancreatic resections in this cohort were associated with significant perioperative morbidity and also impaired pancreatic function in the long-term, the decrease in patient-perceived QoL was only moderate. One might suggest that the majority of patients will accept these constraints for a potential cure of ZES. In particular, MEN1-patients who underwent PD procedures with a high ZES cure rate and low rate of duodenopancreatic reoperations seem to have adapted to their altered gastrointestinal and pancreatic function, leading to a perception of comfort and health close to that of the general population.

The present study has some strengths and some limitations. One strength is the well-defined cohort of prospectively documented MEN1 patients, most of them seen on a regular basis during the annual screening program. Another strength is the unchanged aggressive surgical attitude to indicate surgery for MEN1-ZES during the 30-year study period. Limitations include the retrospective study design with all its known disadvantages. The limited cohort size is underpowered and does preclude strong correlations between the type of duodenopancreatic resections and organ dysfunctions and/or QoL. In addition, the methods of assessing QoL are methodologically biased to some degree since QoL was measured in 2020/2021 for patients operated on at different time points starting from 1987. Furthermore, the very long study period could have influenced the results, due to changes in preoperative diagnostics and operative management. Finally, criteria for a distinct ZES operation have changed over the course of this study, and thus also selection bias cannot be excluded.

In conclusion, perioperative morbidity of duodenopancreatic resections for MEN1-ZES is significant, but not different between PD and non-PD resections. MEN1-ZES should be considered a surgically curable disease, since the cure is over 80% more than 10 years after PD. PD is also preferable compared to non-PD resection, since long-term morbidity as well as QoL are at least equal to non-PD resections. Future prospective international register studies might prove these results, since a prospective RCT is unrealistic given the rarity of the disease.

## 5. Conclusions

Duodenopancreatic gastrinoma in MEN1 should be considered a surgically curable disease. Pancreaticoduodenectomy seems to be the adequate surgical procedure for this disease providing a high long-term cure rate with an acceptable quality of life.

## Figures and Tables

**Table 1:** Clinical characteristics of 35 patients undergoing operation for MEN1-ZES.

	ALL (*n* = 35)	PD (*n* = 11)	Thompson (*n* = 12)	DUODX (*n* = 12)	*p*-Value PD vs. Non-PD *
Gender (female/male)	16/19	6/5	7/5	3/9	0.492
Genetically confirmed	32/35 (91.4%)	10/11 (91%)	12/12 (100%)	10/12 (83.3%)	0.943
Age (years) at ZES surgery (median/range)	42 (30–74)	42 (31–54)	46 (32–55)	44 (40–74)	0.055
Preop. gastrin pg/mL (median/range)	425 (160–44,095)	511 (170–20,746)	425 (170–44,095)	370 (160–693)	0.974
pHPT surgery prior to ZES	33/35 (94%)	10/11 (83%)	12/12 (100%)	11/12 (92%)	0.574
Visualization on imaging	26/35 (74%)	9/11 (82%)	8/12 (67%)	9/12 (75%)	0.504
Duodenal NEN	19/35 (54%)	7/11 (63%)	12/12 (100%)	0/12	0.342
pNEN	3/35 (8%)	3/11 (27%)	0/12	0/12	0.082
Lymph node mets	1/35 (3%)	0/11	0/12	1/12 (8%)	0.507
Other mets	0/35	0/11	0/12	0/12	1

MEN1-ZES = multiple endocrine neoplasia type 1 associated Zollinger–Ellison syndrome; *n* = number; preop = preoperative; pHPT = primary hyperparathyreoidism; mets. = metastases; Thompson = Thompson procedure; DUODX + LAD = duodenotomy, excision of duodenal gastrinomas, and lymphadenectomy; * non-PD resections include both Thompson procedure and DUODX.

**Table 2:** Perioperative and pathological data of patients undergoing operation for MEN1-ZES.

Parameter	ALL (*n* = 35)	PD (*n* = 11)	Thompson (*n* = 12)	DUODX (*n* = 12)	*p*-Value PD vs. Non-PD *
Pts. with complications Dindo ≥ 3	12/35 (37%)	4/11 (36%)	3/12 (25%)	5/12 (42%)	0.433
POPF Typ B	1/35 (3%)	1/11 (9%)	0/12	0/12	0.341
POPF Type C	2/35 (6%)	1/11 (9%)	0/12	1/12 (8%)	0.574
Postoperative bleeding	4/35 (11%)	0/11	2/12 (17%)	2/12 (17%)	0.043
Postoperative pancreatitis	2/35 (6%)	1/11 (9%)	0/12	1/12 (8%)	0.574
Reoperation due to complications	8/35 (20%)	2/11 (18%)	3/12 (17%)	3/12 (25%)	0.667
Perioperative mortality	0/35	0/11	0/12	0/12	1
No. of pNENs in specimen (median/range)	3 (0–18)	4 (0–18)	3 (1–7)	1 (0–3)	0.255
No. duodenal gastrinomas (median/range)	3 (1–13)	3 (1–8)	2 (1–13)	3 (1–6)	0.430
No. resected lymph nodes (median/range)	11 (3–25)	15 (3–25)	14 (8–20)	9 (6–12)	0.279
Lymph node ratio (affected/analyzed)	0.07	0.13	0.07	0.11	0.626
Postop. normal serum gastrin (<125 pg/mL)	30/35 (86%)	10/11 (91%)	10/12 (83%)	10/12 (83%)	0.566
Postoperative Diabetes mellitus #	0/35	0/11	0/12	0/12	1

pNEN = pancreaticoduodenal neuroendocrine neoplasm; PD = pancreaticoduodenectomy; DUODX + LAD = duodenotomy, excision of duodenal gastrinomas, and lymphadenectomy; POPF = postoperative pancreatic fistula; * non-PD includes both Thompson and DUODX resections; # in the first 30 postoperative days.

**Table 3:** Long-term outcomes of surgery for MEN1-ZES.

Parameter	ALL (*n* = 35)	PD (*n* = 11)	Thompson (*n* = 12)	DUODX (*n* = 12)	*p*-Value PD vs. Non-PD *
Median Follow-up, months (range)	134 (6–480)	134 (49–256)	148 (6–480)	69 (14–352)	0.757
Normal Gastrin	14/35 (40%)	9/11 (82%)	2/12 (16%)	3/12 (25%)	0.007
Reoperation for recurrent/persistent ZES	9/35 (26%)	1/11 (9%)	4/12 (33%)	4/12 (33%)	0.080
Reoperation for other pNENs	4/35 (11%)	1/11 (9%)	0/12	3/12 (25%)	0.566
Development of distant gastrinoma metastases	6/35 (17%)	1/11 (9%)	3/12 (25%)	2/12 (17%)	0.353
Development of distant metastases from other NEN	6/35 (17%)	1/11 (9%)	1/12 (8%)	4/12 (33%)	0.353
Insulin-dependent diabetes mellitus	14/35 (40%)	3/11 (27%)	6/12 (50%)	5/12 (41%) #	0.301
Pancreastic enzyme medication	14/35 (40%)	4/11 (36%)	6/12 (50%)	4/12 (33%) #	0.359
Overall survival, months(range)	106 (6–480)	134 (49–256)	148 (6–480)	69 (14–352)	0.757
Disease-free survival, months (range)	61 (0–480)	134 (49–256)	70 (0–480)	44 (0–138)	0.001
QoL-score (EORTC QlC30)	64	76.9	57.4	59.5	0.1963
ZES disease status					
NED	14/35(40%)	9/11 (82%)	2/12 (16%)	3/12 (25%)	<0.001
AWD	18/35(51%)	1/11 (9%)	9/12 (75%)	8/12 (67%)	<0.001
DOD	0/35	0/11	0/12	0/12	1
DURC §	4/35 (11%)	1/11 (9%)	1/12 (8%)	2/12 (16%)	0.777

NED = no evidence of disease; AWD = alive with disease; DOD = dead of disease; DURC = dead of unrelated cause; ZES = Zollinger–Ellison syndrome; pNEN = pancreatic neuroendcocrine neoplasm; * non PD resections include both Thompson procedure and DUODX + LAD; # includes status after reoperations; § includes metastatic thymic carcinoid, metastatic gastric NEC, myocardial infarction, and liver metastasis of a colon carcinoma.

**Table 4:** Quality of life (EORTC QLQ-C30) of patients operated on for MEN1-ZES and other dpNEN at last follow-up.

Scale	ALL- ZES * (*n* = 25)	PD for ZES * (*n* = 9)	DUODX + LAD for ZES * (*n* = 7)	Thompson Procedure for ZES * (*n* = 9)	Non-PD Resections for ZES * (*n* = 16)	*p*-Value (PD vs. Non-PD for ZES *)	PD for Any dpNEN # (*n* = 11)	Non-PD Resection for Any dp-NEN # (*n* = 39)	*p*-Value (PD vs. Non-PD for All dpNEN)	General Population (Hinz 2014)
**Global health/QoL**	**64.6**	**76.9**	**59.5**	**57.4**	**58.3**	**0.1963**	**75.0**	**70.2**	**0.6271**	**75.7**
**Physical functioning**	76.5	83.7	76.2	69.6	72.5	0.4489	84.2	82.3	0.7771	91.0
**Role functioning**	66.5	79.6	64.3	55.6	59.4	0.2327	80.3	76.2	0.9496	88.1
**Emotional functioning**	61.0	61.1	57.1	64.8	61.5	0.8191	58.2	67.6	0.2145	83.2
**Cognitive Functioning**	74.1	79.6	76.2	66.7	70.1	0.9246	77.3	81.9	0.3281	90.5
**Social Functioning**	64.7	72.2	57.1	64.8	61.5	0.7115	72.7	75.5	0.5223	91.5
**Fatigue**	37.1	25.9	39.7	45.7	43.1	0.2772	27.3	30.3	0.8269	19.5
**Nausea and Vomiting**	13.6	9.3	16.7	14.8	15.6	0.7415	9.1	9.6	0.6094	3.1
**Pain**	30.1	16.7	40.5	33.3	36.5	0.1798	16.7	19.3	0.7281	16.5
**Dyspnoea**	20.6	14.8	28.6	18.5	22.9	0.6178	12.1	15.6	0.8309	11.1
**Insomnia**	41.4	33.3	42.9	48.1	45.8	0.5519	30.3	32.6	0.8480	15.7
**Appetite Loss**	19.9	14.8	19.0	25.9	22.9	0.6809	12.1	14.5	0.6274	4.8
**Constipation**	25.2	7.4	28.6	3.7	14.6	0.3472	9.1	12.8	0.6569	5.2
**Diarrhoea**	32.6	37.0	23.8	37.0	31.3	0.6336	36.4	25.5	0.2616	4.9
**Financial Difficulties**	16.2	11.1	19.0	18.5	18.8	0.4134	15.2	17.7	0.9424	5.7

PD resection includes pylorus-preserving and pylorus-resecting partial pancreatectomy; non-PD resections include Thompson procedure, distal pancreatic resections with/without splenectomy, duodenotomy plus LAD, and enucleations; * intention to treat regarding the first operation in case of reoperations; # patients who underwent only 1 duodenopancreatic resection.

**Table 5:** Results of PD for MEN1-ZES (series ≥ 3 patients).

Author/Year	N	Postoperative Normal Serum Gastrin	Median Follow-Up, Years (Range)
Stadil et al., 1993 [23]	3	3 (100%)	0.5–4
Tonelli et al., 2006 [25]	13	10 (77%)	0.5–3
Imamura et al., 2011 [26]	3	3 (100%)	2–10
Lopez et al., 2013 [21]	13	12 (92%)	0.5–11
Santucci et al., 2021 [16]	18	11 (61%)	0–36
Present study	11	9(82%)	4–21
all	61	48 (78.7%)	0–21

## Data Availability

The data presented in this study are available within the article.
